# *Digital Tattoos* in Infectious Diseases Management

**DOI:** 10.1093/ofid/ofag338

**Published:** 2026-06-02

**Authors:** Hideharu Hagiya

**Affiliations:** Department of Infectious Diseases, Okayama University Hospital, Okayama, Japan

**Keywords:** antibiotic allergy, antimicrobial resistance, infectious diseases, isolation, stigma

## Abstract

The widespread adoption of electronic medical records (EMRs) has improved continuity and efficiency in healthcare; however, the permanence of digital documentation may unknowingly and unintentionally disadvantage patients. Certain infectious disease–related labels, particularly those denoting colonization of antimicrobial-resistant organisms, status of stigmatized diseases, or antibiotic drug allergy, frequently persist long after their clinical relevance has expired. These *Digital Tattoos* silently influence clinical decision-making and impose multifaceted burdens, leading to unnecessary isolation, excessive use of broad-spectrum antimicrobials, psychological distress, higher healthcare costs, and reinforcement of stigma. A critical mismatch exists between static (unchanged) EMRs and the dynamic (changeable) nature of clinical conditions. To mitigate the harms of *Digital Tattoos*, we must urgently move beyond passive documentation toward proactive management of healthcare data. Decoupling the permanence of digital records from systemic inequities in patient care hinges on the robust synthesis of *Digital Humility* and patient-oriented risk evaluation.

The development of digital technologies has brought unprecedented, global benefits across many scientific fields. In the healthcare sector, the widespread adoption of electronic medical records (EMRs) has substantially improved data accessibility, continuity of patient care, and clinical efficiency [1]. However, the permanence of digital documentation has simultaneously introduced an underrecognized challenge: certain medical labels, once recorded, may persist far longer than their clinical relevance warrants. These electronic records may have a lasting impact on the attitudes and approaches of healthcare personnel, potentially compromising patient management or limiting access to appropriate treatment [2].

Digital records, especially those capturing a history of contagious diseases and prior antibiotic drug allergy, deserve careful consideration, as they may persist within electronic systems as latent marks that silently shape clinical decision-making beyond their clinical intent. Once labeled in EMRs, documented histories of colonization with antimicrobial resistance (AMR) organisms (eg, methicillin-resistant *Staphylococcus aureus* [MRSA], vancomycin-resistant *Enterococcus* [VRE], extended-spectrum β-lactamase producing *Enterobacterales*, and carbapenem-resistant *Enterobacterales* [CRE]), as well as recorded serological statuses of transmissible viral infections (eg, human immunodeficiency virus [HIV]), may unintentionally function as indelible digital signs.

## WHY IT MATTERS

These labels may outlast their clinical validity, reinforcing stigma and leading to unnecessary isolation or precautionary measures. Also, labeling a patient with a drug allergy history may substantially restrict the future use of antimicrobial agents, thereby resulting in missed opportunities to receive appropriate antimicrobial therapy or undermining antimicrobial stewardship efforts [3, 4]. A concept of “Digital Scarlet Letters” was recently introduced by Bennett et al [5] in the context of sexually transmitted infections, underscoring the imperative for cross-disciplinary attention to the latent socio-technical challenges emerging from digital advancements. Herein, I refer to this issue as *Digital Tattoos* within EMRs, using the term as a metaphor to describe problems that warrant careful recognition and appropriate data management in order to avoid inadvertently disadvantaging patients (Table 1). This manuscript addresses the critical mismatch between static healthcare documentation and the dynamic nature of clinical conditions, followed by the discussion on how to overcome the systemic clinical inertia through proactive data management, facilitated by automated reassessment mechanisms and synchronized de-labeling protocols.

**Table 1.** *Digital Tattoos* in Infectious Disease Management

	*Digital Tattoos*		
Category	AMR Colonization	Stigmatizing Diseases	Antibiotic Drug Allergy
Typical labels/examples	MRSA colonization, positive for ESBL, CRE carriers	Tuberculosis, Hansen's disease, HIV/AIDS	“Penicillin allergy” or “β-lactam allergy”
Purpose of labeling	High-visibility clinical alerts for healthcare providers to implement IPC measures	Rapid identification of sensitive health status	Prevent use of drugs perceived as risky for allergic reactions
Why labels persist	Limited criteria for de-labeling	Limited criteria for de-labeling	Limited criteria for de-labeling
Clinical impact	Unnecessary isolation and contact precautions: delays in necessary medical care	Shaping the “biased clinical gaze” before patient-provider interaction Skewed clinical judgment and unjustified medical isolation	Permanent restricted access to optimal antibiotic therapy
Patient burden	Longer hospital stays, increased healthcare costs, psychological distress, reduced quality of care	Physio- and psychological distress	Higher healthcare costs, increased adverse effects, CDI development, and AMR selection pressure
Driving factors	Mismatch between unchanged EMRs labels and changeable transmission risk Visible alerts amplify risk perception	Historical social stigma and moral prejudice Rigid, context-free health information systems	Allergy labels in EMRs, which persist unless actively reassessed
Suggested approach	Flexibly individualize IPC strategy by patient conditions Establish routine reassessment and structured de-labeling Incorporate automated alerts or expiration parameters for periodic review	De-marginalizing patients by critiquing persistent labels Restoring clinical context and patient agency within digital systems Heightening the moral sensitivity of medical practitioners	De-labeling workflow (skin testing or direct oral challenge) Periodic review and formal de-labeling documentation Ensure cross-system data synchronization to prevent the reappearance of de-labeled entries during record reconciliation

Abbreviations: AIDS, acquired immunodeficiency syndrome; AMR, antimicrobial resistance; CDI, *Clostridioides difficile* infection; CRE, carbapenem-resistant *Enterobacterales*; EMRs, electric medical records; ESBL, extended-spectrum beta-lactamases; HBV, hepatitis B virus; HCV, hepatitis C virus; HIV, human immunodeficiency virus; IPC, infection prevention and control; MRSA, methicillin-resistant *Staphylococcus aureus*.

## *DIGITAL TATTOO* 1: AMR COLONIZATION

First, attention should be directed toward the potential issues associated with the contagiousness of infectious diseases. Labels such as “MRSA colonization,” “Being positive for ESBL-producing Enterobacterales,” or “CRE carrier” are often added to patients’ problem lists or infection control flags with the intention of prompting enhanced infection prevention and control (IPC) measures. Initially, these tags may function effectively as alerts; however, unlike acute diagnoses, they frequently lack standardized criteria for removal or de-labeling. In busy clinical settings, reassessment of such labels is rarely prioritized, allowing outdated infectious disease records to become quasi-permanent facts. Consequently, they may remain visible in EMRs for years or longer, irrespective of microbiological clearance or a reduced risk of transmission, thereby evolving into a lasting digital footprint.

Such persistent labeling for the AMR organisms in the medical records would necessitate unwarranted contact precautions or protracted patient isolation. Preceding evidence suggests that these prophylactic measures can lead to increased healthcare expenditures, delayed clinical procedures, and significant psychological burdens [6–10]. The impact of isolation on the patient experience remains nuanced; while negative outcomes are frequently reported, the perceived burden varies considerably based on clinical context and the quality of provider-patient communication [11].

## STATIC NATURE OF MEDICAL DOCUMENTATION VERSUS DYNAMIC NATURE OF INFECTIVITY

A central paradox lies at the heart of the *Digital Tattoo* problem: a critical mismatch between static (unchanged) medical records and dynamic (changeable) nature of transmission risk of contagious diseases. Binary labels in EMRs—positive or negative, carrier or noncarrier—lack such nuance and fail to capture the variable nature of infectious risks.

To appropriately reduce the clinical burden on healthcare personnel and the potential stigma experienced by patients, IPC strategies should be flexibly individualized to each patient's condition. Historically, transmission-based precautions for AMR pathogens have fluctuated between strictly disciplined universal regulations and more nuanced, risk-stratified approaches [12]. Although current clinical guidelines primarily recommend standardized, systematic protocols over risk-based strategies, the clinical utility and validity of risk-stratified IPC measures are increasingly being investigated [13]. Reflecting this growing body of evidence, significant heterogeneity exists in the implementation of transmission-based precautions aimed at reducing multidrug-resistant organism transmission [14]. Indeed, an online survey indicated that approximately one-third of healthcare facilities in the United States have already discontinued mandatory contact precautions for MRSA and VRE [15]. Notably, another survey found that a substantial majority (77% of physicians and advanced practice providers) expressed a preference for risk-tailored contact precautions for MRSA-colonized patients over universal measures [16]. Collectively, these findings underscore the need to reevaluate uniform and rigid IPC measures for patients colonized with AMR organisms.

To mitigate these risks, EMR systems should integrate intelligent clinical decision support functionalities, such as automated reassessment prompts and defined expiration parameters. For instance, a system could mandate a formal review of an MRSA alert following a series of negative cultures or a specified interval since the last admission. Ultimately, the fundamental challenge posed by *Digital Tattoos* is not the infection control measures themselves, but rather the absence of dynamic reassessment. The static nature of digital records risks perpetuating outdated practices, imposing unnecessary burdens on both patients and healthcare providers.

## *DIGITAL TATTOO* 2: HISTORICALLY STIGMATIZING DISEASES

Patients diagnosed with tuberculosis, Hansen's disease, or human immunodeficiency virus/acquired immunodeficiency syndrome have long faced profound inequities driven by historical social stigma and moral prejudice [17]. Similar patterns of stigmatization are increasingly observed in individuals colonized with AMR organisms [18, 19]; however, the physio- and psychological burden for those affected by these stigmatizing diseases extends far beyond clinical or financial inefficiencies, encompassing profound socio-ethical repercussions. Provided that such labels persist uncritically, they may reinforce inequities by subjecting patients to unjustified isolation and shaping the “clinical gaze” before a provider even meets the patient [20]. Unlike narrative descriptions buried in progress notes, these alerting labels are highly visible and binary, frequently lacking essential clinical details. Worse still, patients themselves have little agency over how long this information remains displayed or how it is interpreted by healthcare personnel.

## DE-MARGINALIZE PATIENTS WITH STIGMATIZING *DIGITAL TATTOOS*

In this manner, the stigmatizing *Digital Tattoos* potentially function as a form of institutionalized prejudice, where the health information system itself dictates an unnecessary care or an increased social distance based on outdated or context-free data. Recent research indicates that this digital labeling of sensitive conditions can foster perceived discrimination, leading to delayed care-seeking behavior, strained patient-provider communication, and suboptimal health outcomes [21, 22].

The stigmatizing implications of *Digital Tattoos* extend far beyond the realm of infectious diseases. Parallel challenges are increasingly documented across diverse clinical domains, most notably in psychiatric diagnoses, substance use disorders, and racial health disparities [23]. In these contexts, persistent and often derogatory digital labels can inadvertently skew clinical judgment, thereby perpetuating systemic biases that undermine the fundamental goal of health equity.

## *DIGITAL TATTOO* 3: ANTIBIOTIC DRUG ALLERGY

Among the various data embedded in EMRs, a documented history of antibiotic drug allergy stands out as one of the most persistent and clinically influential examples of *Digital Tattoos*. These digital tags for previous allergic events are often recorded without confirmatory testing and subsequently persist across decades of care, often irrespective of their clinical relevance and severity [3]. Once inscribed into the problem list, they silently restrict the selection of antimicrobial agents, leading clinicians to refrain from prescribing the drugs concerned without considering the feasibility of their administration.

## FACTS AND CLINICAL CONSEQUENCES OF BETA-LACTAM ALLERGY LABELING

Accumulating evidence clearly demonstrates that most reported penicillin allergies, particularly those originating in childhood, do not represent lifelong hypersensitivity [24]. True IgE-mediated penicillin allergy is well known to wane over time; longitudinal immunologic data indicate that ∼50% of patients lose sensitivity within 5 years and nearly 80% within 10 years [25]. Thus, a substantial proportion of adults carrying a childhood β-lactam allergy label are no longer allergic to the drugs in immunologic point of view. Complementing these observations, consistent evidence indicates that the vast majority of children labeled as β-lactam–allergic—typically on the basis of nonspecific rashes or vague historical symptoms—are actually low risk and tolerate β-lactams when formally evaluated using skin testing or direct oral challenge [26–28]. Despite this robust and reproducible evidence, β-lactam allergy labels frequently persist unchallenged across many healthcare settings.

From a therapeutic perspective, the consequences of these *Digital Tattoos* are profound. Patients labeled as β-lactam–allergic are more likely to receive second-line or broader-spectrum antibiotics, including fluoroquinolones, glycopeptides, or carbapenems, even when β-lactams would be clinically most optimal [29]. This practice is clearly associated with higher healthcare costs and may also be linked to an increased risk of adverse drug events, *Clostridioides difficile* infection, and the emergence of AMR pathogens [25]. Inaccurate allergy labels further undermine antimicrobial stewardship efforts by limiting the use of narrower-spectrum agents and driving increased reliance on last-resort drugs.

## DE-LABELING OF BETA-LACTAM ALLERGY

Conceptualizing β-lactam allergy labels as *Digital Tattoos* reframes them not as static historical facts but as dynamic clinical hypotheses that require periodic validation and assessment. In the digital era, documented records confer durability beyond our intentions; what is entered once may persist indefinitely, reappearing at every admission, prescription orders, or even antimicrobial stewardship sessions. In the absence of structured de-labeling processes, including risk reevaluation and formal documentation, allergy labels in EMRs may persist as clinically obsolete information. Furthermore, a significant technical hurdle is the unintended restoration of deleted labels during medical record reconciliation across different healthcare systems. Even after successful de-labeling, inaccurate allergy records often resurface upon subsequent admissions due to lack of synchronization or uncritical data merging. This highlights the need for a master record approach or more rigorous validation protocols during allergy reconciliation.

In an era increasingly defined by digital permanence, the reassessment of inherited allergy labels should be viewed not as an optional effort but as an essential component of high-quality healthcare management. A recent systematic review underscored the clinical viability of de-labeling, reporting that 95.6% of patients successfully passed oral food challenges despite a documented historical allergy [30]. Furthermore, evidence from a randomized controlled trial indicates that preoperative antibiotic allergy de-labeling is feasible without compromising surgical timelines, ultimately facilitating the administration of optimal β-lactam prophylaxis [31]. Building upon these findings, multidisciplinary de-labeling frameworks have been developed that utilize risk stratification tools to identify appropriate candidates for direct provocation [32]. In this approach, low-risk individuals proceed directly to a graded challenge, bypassing the need for antecedent skin testing.

## COMBAT THE CLINICAL INERTIA BY DIGITAL TECHNOLOGIES

This kind of phenomenon is fundamentally rooted in “Clinical Inertia,” where outdated diagnoses persist within EMRs due to a lack of systematic review and refinement [33, 34]. To counteract this systemic stagnation, a shift toward dynamic documentation is essential, which should be facilitated by automated prompts for de-labeling and the cultivation of *Digital Humility* within healthcare organizations. Recent informatics research has demonstrated the feasibility of embedding clinical decision support tools within EMR to enable real-time risk categorization and seamless integration with de-labeling protocols [35, 36]. For instance, several institutions have successfully integrated an automated best practice alert system into computerized order entry systems [37]. Such interventions encourage clinicians to consider proactive penicillin allergy de-labeling—via direct oral challenge or skin testing—in low-risk populations, transforming static documentation into actionable clinical tasks. This would effectively bridge the gap between historical records and contemporary evidence-based practice.

## MEDICAL BENEFITS OF THE *DIGITAL TATTOOS*

It must be acknowledged, however, that the persistence of digital records serves indispensable clinical functions as well. For instance, prior attention of AMR carriage is a cornerstone of effective antimicrobial stewardship; such inputs inform the selection of appropriate empirical therapy, thereby potentially improving patient outcomes [38]. From a public health perspective, explicit digital labeling is essential during interfacility transfers to mitigate the silent spread of epidemiologically significant pathogens [39]. Furthermore, the premature or unsubstantiated removal of drug allergy labels may pose a significant risk of life-threatening hypersensitivity reactions. Consequently, the primary challenge resides not in the existence of these labels per se, but in their inherent static nature.

The objective in addressing *Digital Tattoos* is not the reflexive deletion of medical history, but rather the implementation of a “life-cycle management” framework in which data are periodically validated and appropriately updated. By ensuring that clinical labels are both visible when contextually relevant and revisable upon the emergence of new evidence, healthcare systems can achieve a critical equilibrium: maintaining clinical safety while fulfilling the imperative to reduce unnecessary stigma and social isolation.

## CALL TO ACTION

*Digital Tattoos*—persistent infectious disease–related inputs in EMRs—are often introduced as precautionary measures, yet they may unknowingly produce multifaceted disadvantages by fostering prejudice, reinforcing stigma, and limiting patients’ access to optimal care. However, recognizing infectious disease labels as potential *Digital Tattoos* should not merely lead to the minimization of digital records. To fully realize the benefits of digital transformation as healthcare systems continue to digitalize, it is essential to give due attention to the latent influence of those medical labels.

**Figure 1.**
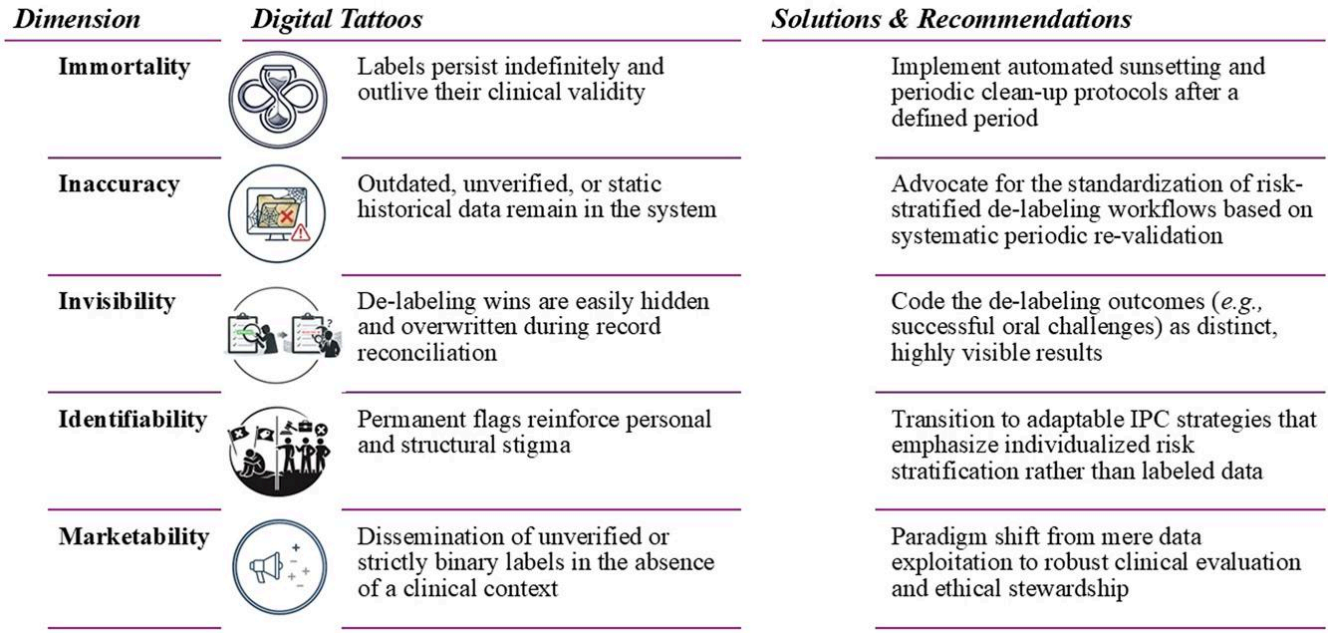
Overcoming *Digital Tattoos* by Addressing the 5 Dimensions of the Digital Health Footprint. This outlines the 5 key dimensions of the digital health footprint—*immortality, inaccuracy, invisibility, identifiability,* and *marketability*—and provides specific interventions to mitigate the harms of persistent medical labels, *Digital Tattoos*. The symbols were produced by the generative AIs. This concept is rooted in the reference [40].

For that, we must pursue responsible and revisable documentation of contagious etiologies and allergic events, addressing the 5 key features of the digital health footprint: invisibility, inaccuracy, immortality, marketability, and identifiability [40]. Healthcare organizations are supposed to address these issues through targeted interventions (Figure 1). To counter “immortality” and “inaccuracy,” healthcare institutes can introduce periodic clean-up protocols for problem lists, whereby nonactive or unverified labels must be re-confirmed or sunsetted after a defined period. To mitigate the risk of data “invisibility,” de-labeling outcomes—such as a successful oral challenge—should be hard-coded as distinct, high-visibility data points. To address “identifiability” and its link to stigma, dynamic IPC strategies should prioritize individualized risk stratification over rigid, permanent flags. Finally, to address the complexities of “marketability,” the paradigm must shift from data exploitation toward robust clinical evaluation and ethical management.

## CONCLUSION

The EMR does not merely store data; it potentially functions as a persistent digital architecture that can propagate structural stigma. Crucially, the impact of clinical labeling varies by condition: whereas AMR-related labels often result in the operational inefficiencies of redundant isolation, labels for conditions like HIV—which require only standard precautions—exert harm through deep-seated structural stigma. Such labeling ultimately compromises the equity and integrity of clinical care.

*Digital Humility*—the recognition that data recorded to protect patients may, over time, outlive their validity and become sources of avoidable harm—needs to be further embraced by healthcare personnel. We must adopt this subtle sense, recognizing that what is documented today may shape patient care long after its relevance has faded. The persistence of outdated clinical labels is a pervasive issue that affects various stages of the healthcare continuum, from acute hospital care to long-term management. Collectively, the current evidence suggests that the adverse consequences of maintaining these *Digital Tattoos* frequently outweigh the clinical risks of de-labeling unverified or erroneous records.
